# Choroid plexus carcinoma in adults: an extremely rare case

**DOI:** 10.11604/pamj.2015.20.302.5854

**Published:** 2015-03-30

**Authors:** Selcuk Ozdogan, Yusuf Emrah Gergin, Sinem Gergin, Ozgur Senol, Mehmet Tiryaki, Necati Tatarli, Tufan Hicdonmez

**Affiliations:** 1 Dr LutfiKirdar Kartal Training and Research Hospital, Istanbul, Turkey; 2 Marmara University Department of Anatomy, Istanbul, Turkey

**Keywords:** Choroid plexus tumors, Choroid plexus carcinomas, Choroid plexus neoplasms in adults

## Abstract

Choroid plexus tumors are rare intraventricular papillary neoplasms derived from choroid plexus epithelium, which account for approximately 2% to 4% of intracranial tumors in children and 0.5% in adults. Almost all choroid plexus carcinomas are seen in children and are extremely rare in adults. Headache, diplopia, and ataxia are the most common symptoms usually caused by mechanical obstruction of cerebrospinal fluid flow followed by hydrocephalus, regardless of tumor location. We present an illustrative case with 73 years old male patient who was consulted with headache to our neurosurgery department. In cranial computed tomography, there was a mass in 4^th^ ventricle and we confirmed the mass with magnetic resonance imaging. After surgery had been performed, pathology specimen was diagnosed as choroid plexus carcinoma which was rarely seen in this age group.

## Introduction

Choroid plexus carcinoma(CPC) is a highly aggressive malignant tumor World Health Organisation Classification(WHO) grade-III that usually presents with cerebrospinal fluid(CSF) obstruction commonly in the lateral ventricles (50%) followed by 4th ventricle (40%), 3rd ventricle (5%), and multiple ventricles (5%) [1]. Almost all choroid plexus carcinomas are seen in children and are extremely rare in adults. Choroid plexus tumors are rare intraventricular papillary neoplasms derived from choroid plexus epithelium which account for approximately 0.5% in adults. The average annual incidence is approximately 0.3 per 1,000,000 population inhabitants. About 80% of CPC arise in children, constituting 20% to 40% of all choroid plexus tumors in this age group [2]. Headache, diplopia, and ataxia are the most common symptoms usually caused by mechanical obstruction of cerebrospinal fluid flow followed by hydrocephalus, regardless of tumor location [3]. Although the occurrence of CPC is higher in childhood, it must be remembered in differentional diagnosis of choroid plexus neoplasms in adults. We present an illustrative case with 73 years old male patient who consult with headache to neurosurgery department. In cranial computed tomography (CT), there was a mass in 4^th^ ventricle and we confirmed the mass with magnetic resonance imaging (MRI).

## Patient and observation

73 year-old male patient was admitted to our clinic complaining of headache. He was examined in clinical followup. In his physical examination, there was no motor deficit and also loss of balance, gait disturbance and sensory deficit were not inspected. Cranial CT was taken after a detailed physical examination and a mass was seen in 4^th^ ventricle. After CT, MRI was performed and presence of mass was confirmed. We performed a cranial surgery with transcortical approach and the mass was completely removed. Pathology specimen was diagnosed as choroid plexus carcinoma which was rarely seen in this age group.

## Discussion

**Figure 1:**
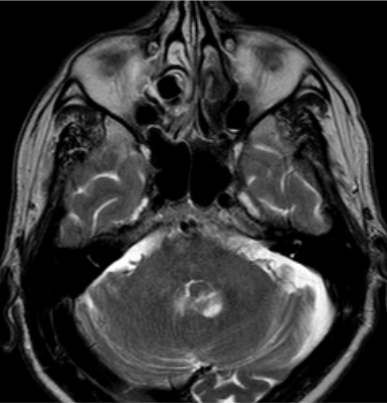
Axial T2 weighted magnetic resonance image of 4th ventricle choroid plexus carcinoma

**Figure 2:**
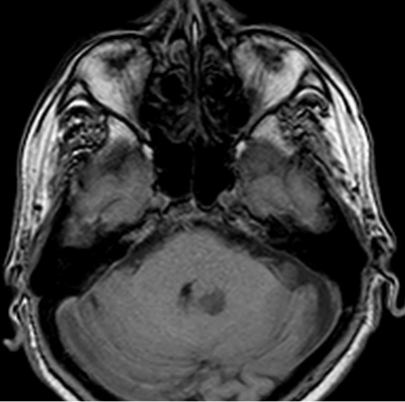
Axial T1 weighted magnetic resonance image of 4th ventricle choroid plexus carcinoma

**Figure 3:**
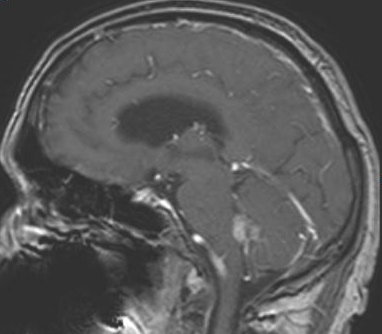
Sagittal contrast enhanced magnetic resonance image of 4th ventricle choroid plexus carcinoma

Choroid plexus tumors are rare intraventricular papillary neoplasms derived from choroid plexus epithelium, which account for approximately 2% to 4% of intracranial tumors in children and 0.5% in adults [2, 4]. Benign papillomas are reported to account for approximately four-fifth of the neoplasms and carcinomas one-fifth. Headache, diplopia, and ataxia are the most common symptoms. Spinal drop metastases are common for CPC and screening of the spine for possible metastasis should be a part of the routine preoperative and postoperative investigation protocol [5]. In our case whole spinal MRI was performed and there was no metastases in any level of spine. Choroid plexus papillomas are being discribed in MRI as lobulated, homogeneous, enhancing masses, whereas carcinomas appear more heterogeneous because of areas of necrosis, calcification or hemorrhage. CT and MRI show iso or hyperdense, T1 isointense, and T2 hyperintense masses that appear enhanced with contrast, within the ventricles, generally associated with hydrocephalus. In our case MRI was reported the mass that hyperintense in T2 weighted (Figure 1), isointensein T1 weighted (Figure 2) and contrast enhanced (Figure 3). There was minimal hydrocephalus in cranial CT but there was no diagnostic criteria about hydrocephalus [1, 6].

Macroscopic investigations of CPC are solid, hemorrhagic and necrotic. Histopathological results of CPC (WHO grade III) are mitoses, nuclear pleomorphism, increased cellular density, blurring of the papillary growth pattern and necrosis. Histological grading is recognized as an important prognostic factor in choroid plexus tumors and also affects the decision toward adjuvant radiotherapy and chemotherapy. On immunohistochemical staining, it was found that the tumor was positive for S-100, cytokeratin and glial fibrillary acidic protein(GFAP). In our case; during the surgery the mass was look like hemorrhagic and solid but also a cystic part was seen. Some necrotic parts were identified. Differential diagnosis of an intraventricular papillary tumor includes several possibilities. Villous hypertrophy of the choroid plexus, papillary variant of ependymoma, myxopapillary ependymoma, papillary meningioma. In infants, primitive neuroectodermal tumors (PNET), atypical teratoid or rhabdoid tumor, metastatic neoplasms being renal cell carcinoma could be reported [3, 7]. Surgical resection is considered to be the most effective treatment for CPC but patients treated only with surgery have had a very poor outcome. Most of the disease progresses rapidly and patients often die within 1 year. The early use of radiation therapy may extend the survival. Chemotherapy contributes to long-term survival, but it can not prevent recurrence [8]. Bleggi-Torres et al. reported 15 cases of CPC and the oldest patient of his series is 22 years old [9]. Main symptoms are hydrocephalus, intracranial hypertension and convulsion in the series. Wrede et al. reported a meta-analysis of 347 CPC patients (children and adults) up to 2004 and they investigated that second surgery make the survival better [10].

## Conclusion

Choroid plexus carcinomas must be remembered within the differential diagnosis of all intraventricular neoplasms in adults. Total excision by surgery, radiotherapy and chemotheraphy are the effective treatment modalities for choroid plexus carcinomas.
